# Proton therapy with a fixed beamline for skull-base chordomas and chondrosarcomas: outcomes and toxicity

**DOI:** 10.1186/s13014-021-01961-9

**Published:** 2021-12-20

**Authors:** Konstantin Gordon, Igor Gulidov, Sergey Koryakin, Daniil Smyk, Tatyana Makeenkova, Danil Gogolin, Olga Lepilina, Olga Golovanova, Alexey Semenov, Sergey Dujenko, Kira Medvedeva, Yuri Mardynsky

**Affiliations:** 1 Department of Proton Therapy, A. Tsyb Medical Radiological Research Centre (MRRC), 4 Korolev Street, Obninsk, Russia 249036; 2 Department of Histology, Cytology and Embryology, Medical Institution, People’s Friendship University of Russia, Moscow, Russia; 3 Department of Radiophysics, A. Tsyb Medical Radiological Research Center, Obninsk, Russia

**Keywords:** Proton therapy, Fixed beam, Chordoma, Chondrosarcoma, Pencil beam, IMPT

## Abstract

**Aim:**

This study presents an analysis (efficacy and toxicity) of outcomes in patients with skull-base chordomas or chondrosarcomas treated with a fixed horizontal pencil proton beam.

**Background:**

Chordomas (CAs) and chondrosarcomas (CSAs) are rare tumours that are usually located near the base of the skull and very close to the brain's most critical structures. Proton therapy (PT) is often considered the best radiation treatment for these diseases, but it is still a limited resource. Active scanning PT delivered via a fixed pencil beamline might be a promising option.

**Methods:**

This is a single-centre experience describing the results of proton therapy for 31 patients with CA (n = 23) or CSA (n = 8) located near the base of the skull. Proton therapy was utilized by a fixed pencil beamline with a chair to position the patient between May 2016 and November 2020. Ten patients underwent resection (32.2%), 15 patients (48.4%) underwent R2 resection, and 6 patients had unresectable tumours (19.4%). In 4 cases, the tumours had been previously irradiated. The median PT dose was 70 GyRBE (relative biological efficacy, 1.1) [range, 60 to 74] with 2.0 GyRBE per fraction. The mean GTV volume was 25.6 cm^3^ [range, 4.2–115.6]. Patient demographics, pathology, treatment parameters, and toxicity were collected and analysed. Radiation-induced reactions were assessed according to the Common Terminology Criteria for Adverse Events (CTCAE) v 4.0.

**Results:**

The median follow-up time was 21 months [range, 4 to 52]. The median overall survival (OS) was 40 months. The 1- and 2-year OS was 100%, and the 3-year OS was 66.3%. Four patients died due to non-cancer-related reasons, 1 patient died due to tumour progression, and 1 patient died due to treatment-related injuries. The 1-year local control (LC) rate was 100%, the 2-year LC rate was 93.7%, and the 3-year LC rate was 85.3%. Two patients with CSA exhibited progression in the neck lymph nodes and lungs. All patients tolerated PT well without any treatment interruptions. We observed 2 cases of ≥ grade 3 toxicity, with 1 case of grade 3 myelitis and 1 case of grade 5 brainstem injury.

**Conclusion:**

Treatment with a fixed proton beam shows promising disease control and an acceptable toxicity rate, even the difficult-to-treat subpopulation of patients with skull-base chordomas or chondrosarcomas requiring dose escalation.

## Introduction

Chordomas and chondrosarcomas are rare among malignancies and mainly affect the skull base, sacrum bones, and vertebral column (less). These tumours arise from either notochordal remnants or mesenchymal cells. Because of their critical location and high local recurrence rate, even though they have a low risk of metastasis, skull-base CA and CSA are challenging to treat [1]. Their management requires a multidisciplinary approach. As gross tumour resection is not achievable in the majority of cases, RT is of vital importance. Moreover, a high total dose (≥ 70 Gy) is needed to achieve adequate tumour control. Photon therapy, the most widely available irradiation treatment, usually shows inferior clinical outcomes due to the inability to strike a balance between delivering an effective dose and respecting normal tissue tolerance.

Historically, these malignancies (CA and CSA) were the first targets for proton therapy (PT) [2]. In 1999, Munzenrider et al. published the largest series of CA/CSA patients treated with PT in Massachusetts General Hospital to date, consisting of 519 cases. The 5-year results showed local relapse-free rates of 73% for CA and 98% for CSA [3]. Another remarkable article from Loma-Linda University outlined two necessary treatment volumes, high- and low-risk, which is now mandatory used for CA/CSA target contouring [4].

Nevertheless, most of the published research describes clinical experiences related to passive-scattering protons [5]; however, active scanning techniques (e.g., spot or pencil beam) allow for better conformality, literally by “painting” the target volume and sparing surrounding normal tissues. These features help to respect dose constraints better, even in tumours located at the base of the skull, which require high doses. Compared to recent intensity-modulated photon techniques, passive-scattering PT no longer confers the advantages of efficient dose distribution; in addition, it requires specific hardware and produces contaminating secondary neutrons.

Although there is a forecast to increase the number of particle facilities in Europe to 45 centres by 2023, the use of this irradiation treatment is still infrequent [6]. Despite recent technical advances, the primary obstacles to widespread implementation are PT cost and the deficit of gantry-equipped centres. Chair-based treatment with horizontal fixed beams can be used for many disease locations, with skull bases being among them, and thus warrants further attention [7].

Since 2015, we have exclusively treated patients with the fixed pencil-beam scanning (PBS) system, the first installed in Russia [8]. We represent our initial experience with PT for CA/CSA patients, focusing on the clinical outcomes and toxicity of the treatment. The survival rate, disease control, and PT-related adverse events were analysed.

## Materials and methods

Thirty-one patients diagnosed with CA or CSA located at the base of the skull who were treated with proton therapy at A. Tsyb Medical Radiological Research Center between May 2016 and November 2020 were identified and approved for inclusion in a retrospective analysis by a local institutional review board. Informed consent was waived due to the retrospective nature of this study. All patients were 18 years old or older with histologically or radiologically confirmed primary tumour or evidence of relapse. Eight patients were treated for recurrence after prior surgical or combined treatment, with 4 patients undergoing re-irradiation. Twenty-three patients (74.2%) had CA, and 8 patients (25.8%) had CSA. Of the 8 patients with CSA, 62.5% had grade 2 disease, and 37.5% had grade 1 disease. Most of the patients had ECOG (Eastern Cooperative Oncology Group) status 1 [0–3]. The patient and tumour characteristics are summarized in Table 1.

**Table 1:** Patient and treatment characteristics

Patient characteristics	Number (or %)
Total patients	31
Median follow-up time in months	21 [4–52]
*Gender*	
Female	20 (64.5%)
Male	11 (35.5%)
Median age in years	50 [27–71]
Median ECOG status	1 [0–3]
*Histology/Radiological diagnosis*	
Chordoma	23 (74.2%)
Chondrosarcoma	8 (25.8%)
*Surgery*	
Unresectable	6 (19.4%)
R1	10 (32.2%)
R2	15 (48.4%)
*Indication for irradiation*	
Primary	6 (19.4%)
Adjuvant	18 (58.0%)
Progression/recurrence (including re-RT)	7 (22.6%) / 4 (12.9%)
*Critical OAR involvement*	
Brainstem	6
Visual pathway	7
Spinal cord	1
Cranial nerves	8
Hypophysis	10
*Involved anatomical site*	
Clivus	26
Pyramids	3
Ethmoidal bone	4
Orbit	1
Cervical spine	3
Nasal cavity	3
*Symptoms*	
Headache	15
Visual disfunction (diplopia)	10
Facial paresis	2
Motion paresis	3
Otalgia	3
Ptosis	2

### Proton therapy

Proton therapy was utilized with a fixed horizontal proton beam in a seated position, supported by cone-beam computer tomography (CT) and standard mask immobilization [9]. Diagnostic pre- and postoperative magnetic resonance images (MRI) (sequences: T_1_—1 mm thickness, T_1_ contrast—1 mm, T_2_—3 mm, Flair—3 mm, Fat-suppressed—3 mm) were coregistered for 1 mm CT simulation scans for optimal tumour and/or tumour bed and organ at risk (OAR) delineation. Gross tumour volume (GTV) was defined as macroscopic rest tumour or tumour bed (for R1 surgical margins). The clinical target volume (CTV) consisted of the preoperative disease extent, residual tumour defined by CT and MRI, and 10 mm margin at the area at risk anatomically adapted to the natural borders. For the first planning target volume (PTV1) generation, the corresponding CTV was expanded by a 3-mm margin, and a dose of 50 GyRBE was prescribed. Consequent PTV2 was created from the GTV, and the patient received as high a dose as possible considering the tolerance of surrounding critical structures. All delineations were cross-checked by senior physicians with a Ph.D.

The median total dose in our group was 70 GyRBE, which is sufficient for adequate tumour control. In the 3 patients who received re-irradiation, we treated PTV2 only, and for 1 patient, we performed the whole scheme with PTV1/PTV2 due to the low dose (40 Gy) and interim (15 years) from prior RT. In these cases, we used 60–66 GyRBE due to the known limits of re-irradiation. Serial OARs (i.e., spinal cord, optic nerves, chiasma, and brainstem) were allowed to receive a cumulative dose from both courses < 120–125% from their initial constraints. Delineation of the OAR and limit prescription was performed and chosen following the recommendations by Scoccianti S. et al. [10].

**Fig. 1:**
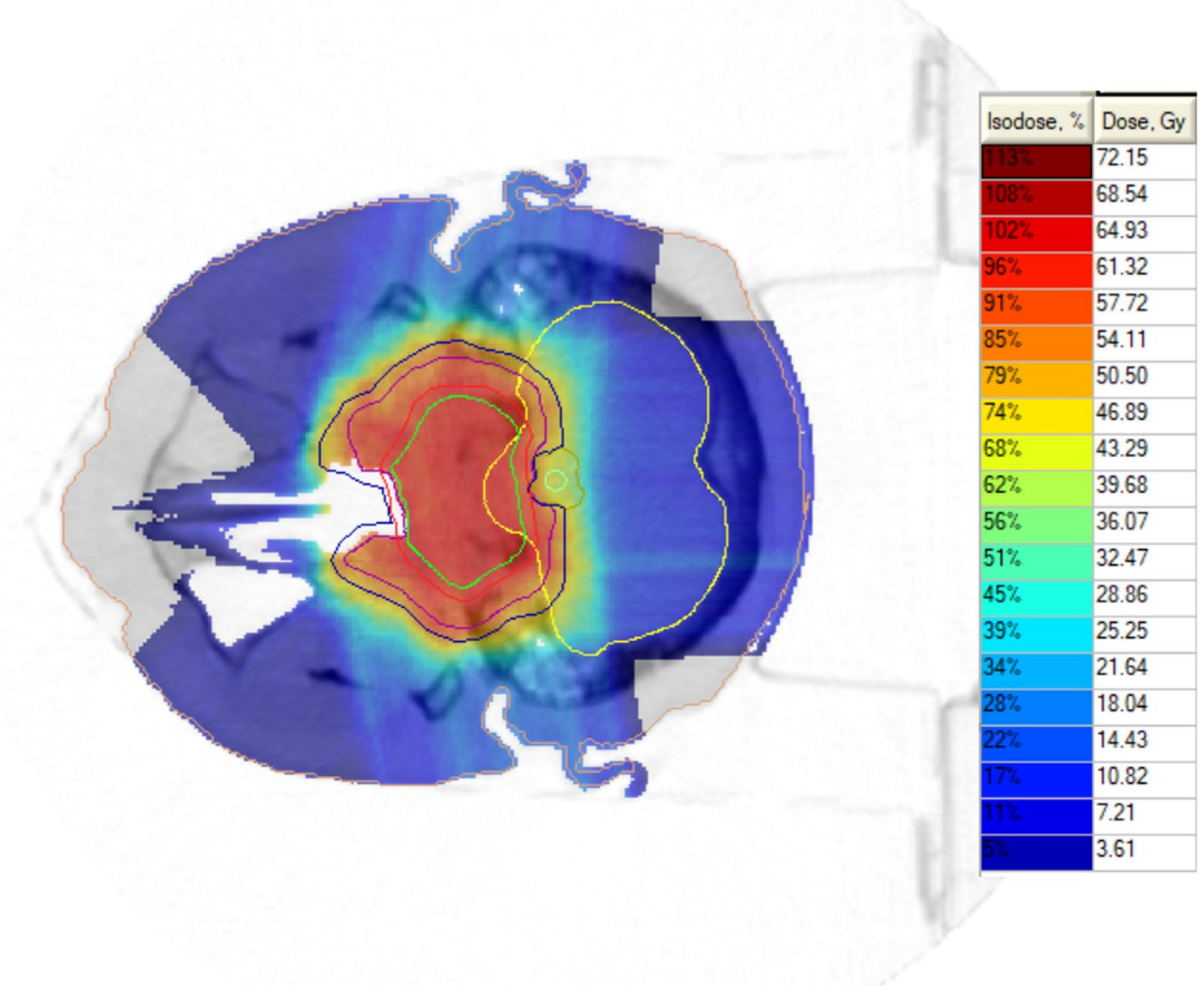
Representative proton irradiation summarized plan for skull-base CA: PTV1 (dark blue line) + PTV2 (red line). Tumor volume (green contour) = 30.6 cm^3^ (in legends physical doses are given)

**Fig. 2:**
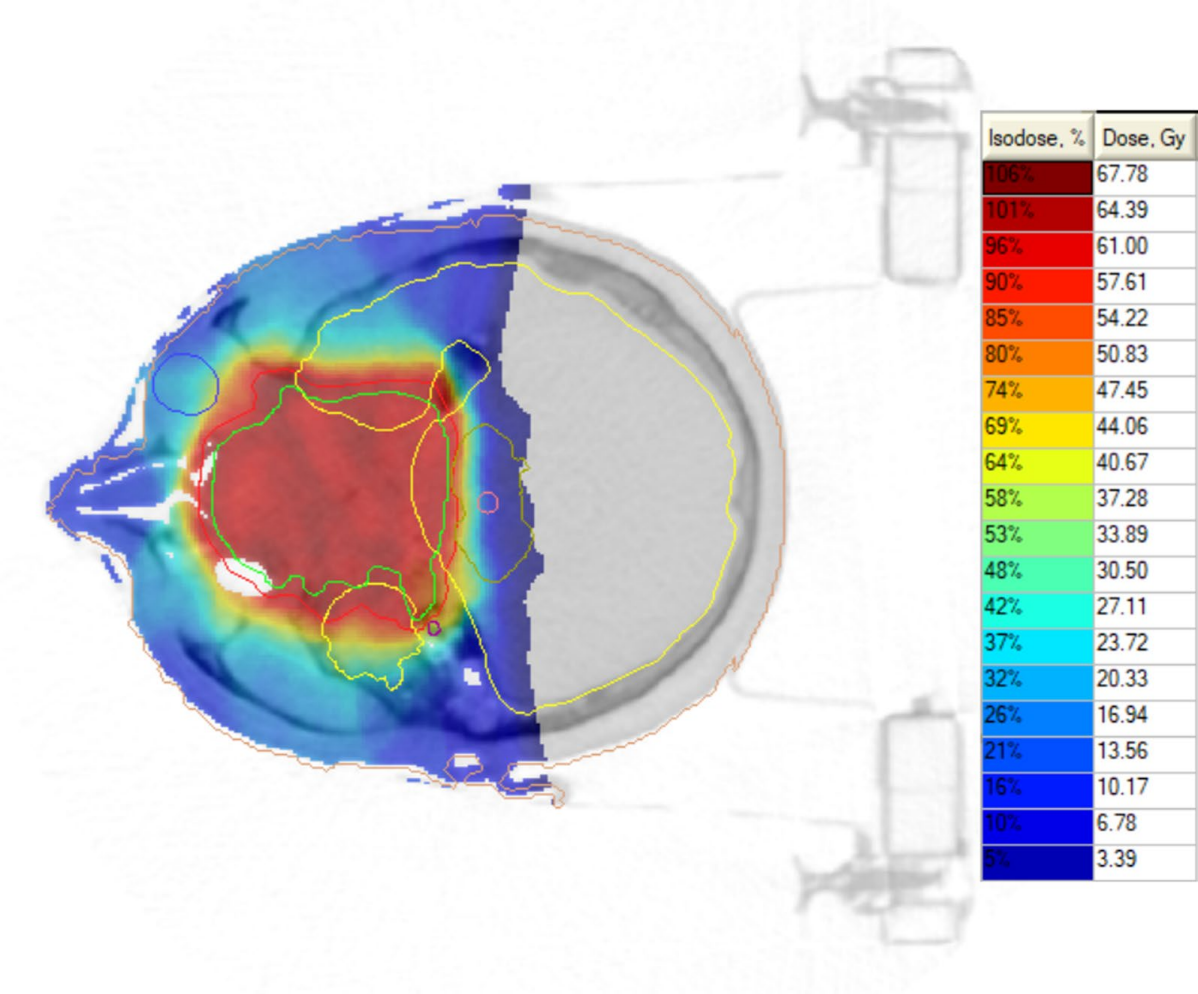
Representative proton irradiation summarized plan for giant unresectable skull-base CA: PTV1 (red line). Tumor volume (green contour) = 120.8 cm^3^ (in legends physical doses are given)

Treatment was delivered in a conventional regimen, with intensity-modulated proton therapy (IMPT) based on a pencil-beam fixed line, with a 360° rotating chair to position the patient. Session accuracy was ensured via 3D cone-beam CT scans and portal imaging before each field. Planning and treatment (Monte Carlo-based) were performed with the help of the Prometheus PT complex (JSC Protom, Russia). We used multifield optimized PTV-based plans, usually generated with 5–6 fields. The PT dose was prescribed to the PTV with the aim of at least 95% coverage, but in the case of meeting OAR constraints, dose limits usually prioritized target coverage depending on the individual clinical situation. Table 2 depicts the PT parameters and planning results, and Figs. 1 and 2 show the representative proton (IMPT) plans.

**Table 2:** Proton therapy parameters

PT dosimetry	Number (median, range)
Median GTV volume in cm^3^	25.6 [4.2–115.6]
Median D95	97.8 [89.2–100.0]
Median proton dose (GyRBE)	70.0 [60.0–74.0]
Dose per fraction	2 GyRBE
Number of fractions	35 [30–37]
*Dose to OARs*	
Brain stem (Dmax, GyRBE)	52.85 [13.6–64.8]
Chiasma (Dmax, GyRBE)	45.9 [0.4–66.9]
Optical nerve right (Dmax, GyRBE)	43.2 [0–66.5]
Optical nerve left (Dmax, GyRBE)	44.8 [2–63.5]
Spinal cord (Dmax, GyRBE)	20.4 [0–50.9]
Temporal lobe right (Mean dose, GyRBE)	18.0 [15.0–28.0]
Temporal lobe left (Mean dose, GyRBE)	16.5 [14.0–29.3]
Hypophysis (Mean dose, GyRBE)	52.1 [32–69.8]

### Statistical analysis

Study endpoints were to estimate actuarial overall survival (OS), local control (LC), and toxicity rates. Survival values were calculated using the Kaplan–Meier method with the analysis made using GraphPad Prism 8. As our study group contained a limited number of cases, we decided not to categorize patients by pathology or treatment parameters since small groups' results are difficult to assess correctly.

### Clinical results and toxicity

The median follow-up time from proton irradiation was 21 months [range, 4 to 52]. The median survival time was 40 months, the 1- and 2-year OS rates were 100.0%, and the 3-year OS rate was 66.3%. Six patients died by the time of analysis, in 4 cases due to non-tumour-related reasons. One patient died due to tumour progression 27 months after PT, and 1 died due to late radiation-induced brainstem toxicity despite the tolerance only being exceeded slightly [D max = 64.8 GyRBE]. In that case, we thought PT would be a palliative treatment because the tumour almost entirely enveloped the brainstem, and the patient's ECOG was 3. Nevertheless, PT helped this patient survive two years, with injury occurring 22 months after PT. Despite treatment, which included bevacizumab, necrosis resulted in the patient's death at 25 months after PT.

Another 2 patients died from coronaviral pneumonia, 1 patient died due to acute gastric vessel blow-out, and 1 patient died due to heart infarct.

The 1-year LC was 100.0%, the 2-year local control (LC) was 93.7%, and the 3-year LC was 85.3%. Two patients with CSA also had locoregional progression in neck lymph nodes (1) and distant lung metastasis (1). They received surgery and chemotherapy and were alive without any signs of further progression at the time of the analysis.

During treatment, all patients tolerated PT well, without any course gaps. Acute toxicity was represented by grade 1 (n = 5, 16.1%) or grade 2 (n = 1, 3.2%) keratitis and laryngeal mucositis grade 2 (n = 3, 9.7%). Most events were recorded 3–6 months after finishing PT. Late toxicity was present with grade 2 temporal lobe necrosis (n = 2, 6.4%), grade 1 xerostomia (n = 1, 3.2%) and grade 2 persistent headache (n = 4, 12.9%) developing > 12 months after irradiation.

Additionally, we observed 2 cases of ≥ grade 3 late toxicity (6.4%), namely, 1 case of grade 3 myelitis (11 months since the PT) and 1 case of grade 5 brainstem injury. The patient with grade 3 myelitis had a metal spinal fixation system, with range uncertainties due to image artefacts and density. The injury was controlled with the help of corticosteroids and bevacizumab and in 2 cases of temporal lobe necrosis. We did not observe an increase in already persistent neurological symptoms. Most patients (80.6%) reported a subjective improvement in these symptoms after treatment. Following linear energy transfer (LET), assessment of treatment plans did not show a correlation between high-LET points and necrotic areas, excluding the patient with brainstem necrosis.

## Discussion

The primary issue of CA and CSA is local recurrence, as it determines the intensity and optimization of local therapy options such as surgery and radiotherapy. Due to known anatomical reasons, treatment of these diseases remains a complicated problem in neurooncological science, with limited surgical options available for the skull-base site. This issue has led high-dose radiation therapy to become an essential treatment with curative intent, especially after incomplete resection (or for unresectable tumours) [11].

Herein, we present our results of using PT via fixed horizontal PBS in 31 patients with skull-base CA or CSA. With a median follow-up time of 21 months, we achieved 100% 2-year OS and 93.7% 2-year LC. The three-year LC was 85.3%, and the three-year OS was 66.3%, with most patients (n = 4) dying due to non-cancer-related reasons. The toxicity of PT in our cohort was moderate, and we recorded only 2 cases (6.4%) of serious adverse events, with one treatment-related death. This was a predictable outcome due to the poor condition of the patient and deep infiltration of the brainstem before therapy. Another radiation-induced adverse reaction was grade 3 myelitis in a patient with spinal metal construction, which always causes uncertainty in PT planning [12]. Generally, most other radiation-related events were noncritical and manageable.

Conventional photon therapy is prevalent in cancer treatment due to its accessibility and cost. Current technologies such as intensity modulation or volumetric arc therapy can achieve good results with acceptable toxicity for most tumour locations. Nevertheless, in the case of tumours located at the base of the skull, external-beam therapy with conventional photons might not be able to physically achieve an adequate total dose (≥ 70 Gy) without increasing the risk of damage to OARs. Indeed, with photon therapy, the 5-year local control or progression-free rates are usually reported to range from 15 to 66%, as the total target doses are lower than what is needed [13].

Despite the fact that Munzenrider et al. showed combined photon-proton treatment results with a passive-scattering beam, the authors reported positive clinical outcomes [3]. Toxicity was not the primary study endpoint in this series; only 3 patients died due to severe brainstem injury, and another 8 had ≥ grade 3 toxicity. These excellent results were recently supported by other authors [4, 14–16].

Proton therapy can be delivered in two main ways: passive scattering (when protons are spread out by using scattering foils and conformed laterally with apertures) or, the most advanced technique, active scanning PT. This method is flexible, with a small proton beam generated and varied to treat different spots and layers in the target area. Intensity modulation became a widely used form of PT, with newly constructed centres usually equipped with such capability. However, according to the Particle Therapy Co-Operative Group statistics, nearly one-third of centres are equipped with gantry and pencil-beam systems [17]. Although it has undoubted clinical advantages, the bulk and cost of gantry systems limit the number of available PT centres [18].

Recent studies have shown that fixed beamlines can be effective from both a physical and economic point of view [19]. In 2020, Fabiano et al. presented a treatment bunker with a fixed-beam PT line and 3D-linac inside [20]. The authors revealed the optimal combination of protons and photons for head and neck cancers. However, in a lying position, a fixed beamline might limit field delivery possibilities [21]. A rotating chair can be a solution, showing promising outcomes in head and neck locations, for example [22, 23].

In 2017, Hall et al. defined tumours at the base of the skull as the targets that benefit the most from PT [24]. Treatment with an active scanning beam reduces the toxicity and can potentially increase efficacy. In 2009, Ares et al. reported outcomes of spot-scanning PT for 64 skull-base CA/CSA patients [25]. With a mean follow-up time of 38 months, the 5-year LC rates were 81% for CA and 94% for CSA. Brainstem compression (*p* = 0.007) and GTV > 25 cm^3^ (*p* = 0.03) were found to be risk factors for lower LC rates. The actual 5-year freedom from high-grade toxicity was 94%. In our group, we had boundary tumour volume (median GTV 25.6 cm^3^), and the rate of early local control (1 and 2 years) was > 93%. Nevertheless, over a longer period, LC decreased to 85.3% (3-year LC). Obviously, this was a result of the balance between optimal dose coverage for large tumours and the sparing of OARs.

Grosshans et al. in 2014 reported a small series of 15 patients with CA or CSA treated with spot-scanning PT [26]. Only 1 case of local recurrence and 1 case of distant failure were reported, and no cases of > grade 2 toxicity were reported over a median follow-up time of 27 months.

In 2016, Feuvret et al. described a series of 159 patients with skull-base CA treated with a combination of photons and protons [15]. In 126 patients, a fixed beamline for the PT step was used, and only for 23 cases was PT delivered with the help of a gantry system. Nevertheless, the authors achieved favourable results: the 5- and 10-year survival rates were 96.4% and 93.5%, respectively, and the toxicity rate was low (a median follow-up time was 77 months). Data from the US National Cancer Data Base on CA and CSA were reported in 2019 by Palm et al. [27]. Treatment outcomes of 863 CSA patients and 715 CA cases were analysed, with 234 patients being treated with proton therapy. Proton therapy was identified as a significant factor for better outcomes in both malignancies. Unfortunately, the authors did not divide the PT group based on passive scattering and active scanning beams.

The most recent article on active scanning PT for skull-base CA and CSA was published in 2021. Parzen et al. presented their experience of IMPT with simultaneous-integrated boost delivered via gantry machine in 13 patients with small GTV volumes (median 3.4 cm^3^) [28]. With a median dose to the GTV/CTV of 72.3/50.4 GyRBE, 100% OS and LC were recorded after a median follow-up time of 10.7 months. The absence of > grade 2 toxicity events was reported, although there was 1 report of grade 2 temporal lobe necrosis.

In the recent National Comprehensive Cancer Network guidelines, CA and CSA were described as preferred targets for proton therapy [29]. Unfortunately, access to this technology, especially in developing countries, is still relatively low. Although our study cohort is limited due to the retrospective nature of this work and the inhomogeneous group of patients included, we achieved promising outcomes and report only moderate toxicity using a fixed PBS proton machine (which is typical compared to the gantry system) for a very challenging-to-treat subpopulation of patients with skull-base CA or CSA. Compared to the abovementioned articles on PT for skull-base CA and CSA, we report noninferior clinical results for our patients, the majority of whom have unfavourable tumour volumes.

## Conclusion

Pencil beam proton therapy via a fixed horizontal beamline is a promising treatment for skull-base chordomas and chondrosarcomas. Our data suggest that PT is effective and safe for these malignancies, so patients with CA or CSA can be recommended for treatment at centres offering this specialized treatment without gantry systems.

## Data Availability

The data used to support the findings of this study is restricted by the Ethical Committee of A.Tsyb MRRC in order to protect patients privacy. Data is available upon request from the corresponding author for researchers, who meet the criteria for access to confidential data.

## Abbreviations

CA: Chordoma
CSA: Chondrosarcoma
CT: Computer tomography
CTCAE: Common terminology criteria for adverse events
CTV: Clinical target volume
ECOG: Eastern cooperative oncology group
Fx: Fraction
GTV: Gross tumor volume
Gy: Gray
JSC: Joint stock company
IMPT: Intensity-modulated proton therapy
LC: Local control
LET: Linear energy transfer
MRI: Magnetic resonance imaging
OAR: Organ at risk
OS: Overall survival
PBS: Pencil-beam scanning
PT: Proton therapy
PTV: Planning tumor volume
RBE: Relative biological efficacy
RT: Radiotherapy
US: United States
